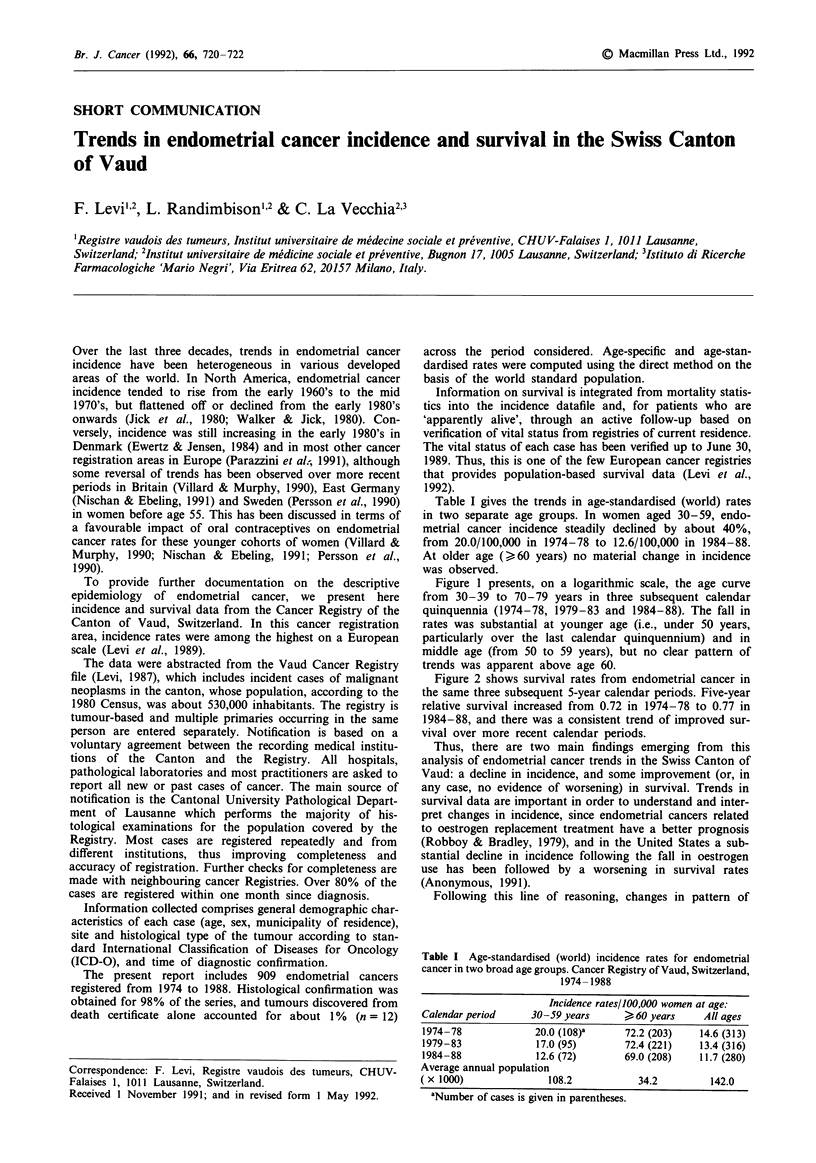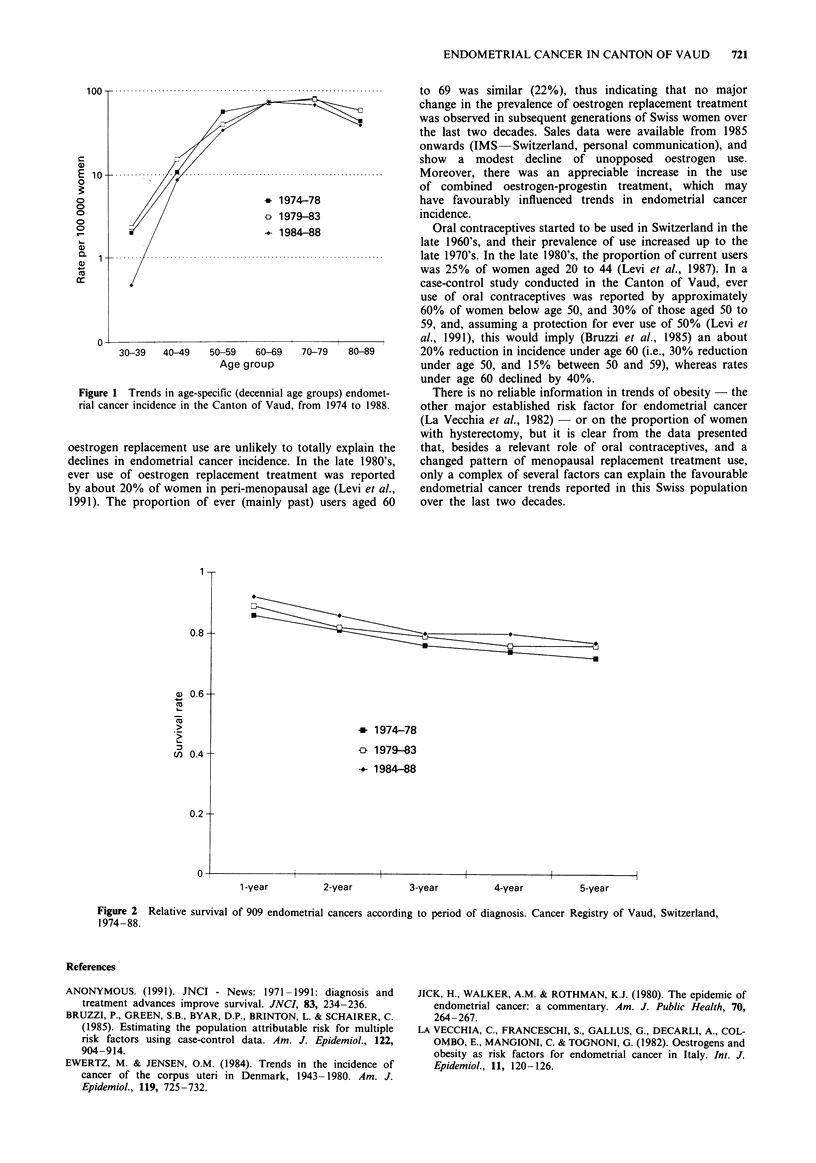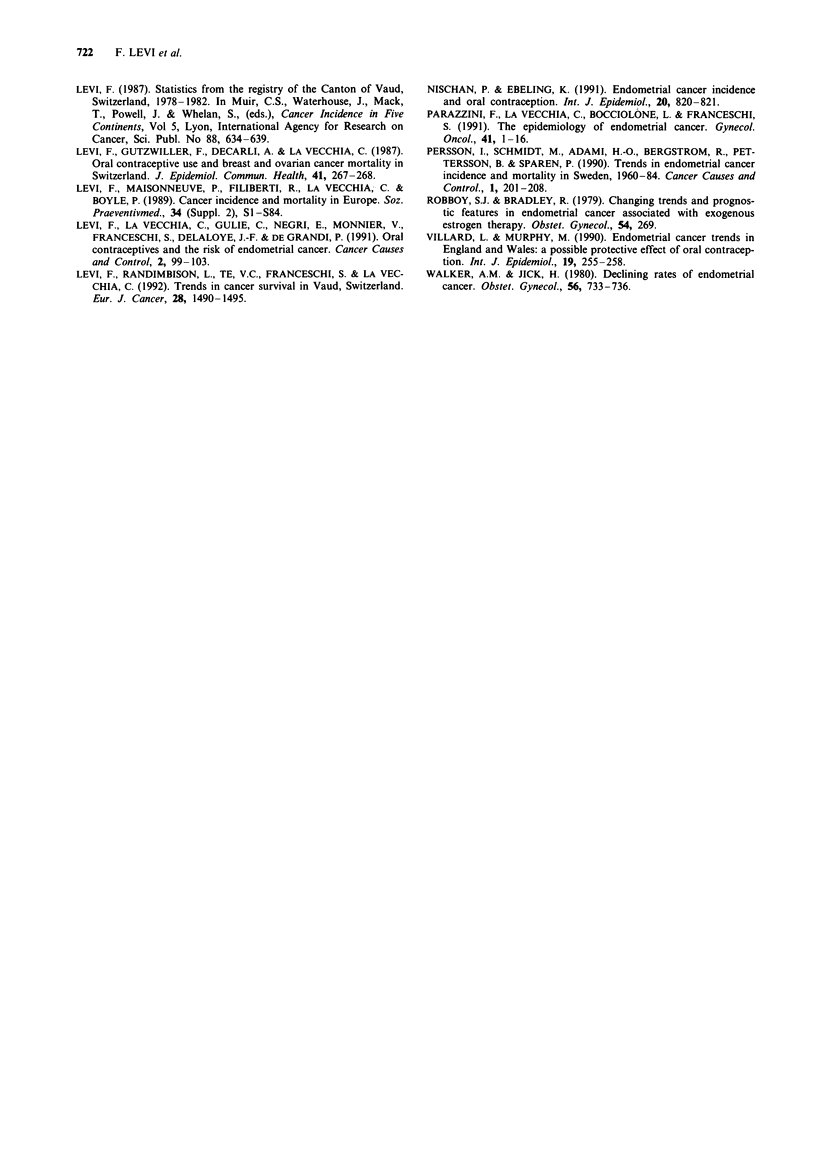# Trends in endometrial cancer incidence and survival in the Swiss Canton of Vaud.

**DOI:** 10.1038/bjc.1992.345

**Published:** 1992-10

**Authors:** F. Levi, L. Randimbison, C. La Vecchia

**Affiliations:** Institut universitaire de médecine sociale et préventive, CHUV-Falaises, Lausanne, Switzerland.


Br. J. Cancer (1992), 66, 720-722 © Macmillan Press Ltd., 1992

SHORT COMMUNICATION

### Trends in endometrial cancer incidence and survival in the Swiss Canton of Vaud

F. Levi[1,2], L. Randimbison[1,2] & C. La Vecchia[2,3]

[1]*Registre vaudois des tumeurs, Institut universitaire de médecine sociale et préventive, CHUV-Falaises 1, 1011 Lausanne, Switzerland;* [2]*Institut universitaire de médicine sociale et préventive, Bugnon 17, 1005 Lausanne, Switzerland;* [3]*Istituto di Ricerche Farmacologiche 'Mario Negri', Via Eritrea 62, 20157 Milano, Italy.*

Over the last three decades, trends in endometrial cancer incidence have been heterogeneous in various developed areas of the world. In North America, endometrial cancer incidence tended to rise from the early 1960's to the mid 1970's, but flattened off or declined from the early 1980's onwards (Jick *et al.*, 1980; Walker & Jick, 1980). Conversely, incidence was still increasing in the early 1980's in Denmark (Ewertz & Jensen, 1984) and in most other cancer registration areas in Europe (Parazzini *et al.*, 1991), although some reversal of trends has been observed over more recent periods in Britain (Villard & Murphy, 1990), East Germany (Nischan & Ebeling, 1991) and Sweden (Persson *et al.*, 1990) in women before age 55. This has been discussed in terms of a favourable impact of oral contraceptives on endometrial cancer rates for these younger cohorts of women (Villard & Murphy, 1990; Nischan & Ebeling, 1991; Persson *et al.*, 1990).

To provide further documentation on the descriptive epidemiology of endometrial cancer, we present here incidence and survival data from the Cancer Registry of the Canton of Vaud, Switzerland. In this cancer registration area, incidence rates were among the highest on a European scale (Levi *et al.*, 1989).

The data were abstracted from the Vaud Cancer Registry file (Levi, 1987), which includes incident cases of malignant neoplasms in the canton, whose population, according to the 1980 Census, was about 530,000 inhabitants. The registry is tumour-based and multiple primaries occurring in the same person are entered separately. Notification is based on a voluntary agreement between the recording medical institutions of the Canton and the Registry. All hospitals, pathological laboratories and most practitioners are asked to report all new or past cases of cancer. The main source of notification is the Cantonal University Pathological Department of Lausanne which performs the majority of histological examinations for the population covered by the Registry. Most cases are registered repeatedly and from different institutions, thus improving completeness and accuracy of registration. Further checks for completeness are made with neighbouring cancer Registries. Over 80% of the cases are registered within one month since diagnosis.

Information collected comprises general demographic characteristics of each case (age, sex, municipality of residence), site and histological type of the tumour according to standard International Classification of Diseases for Oncology (ICD-O), and time of diagnostic confirmation.

The present report includes 909 endometrial cancers registered from 1974 to 1988. Histological confirmation was obtained for 98% of the series, and tumours discovered from death certificate alone accounted for about 1% ($n = 12$) across the period considered. Age-specific and age-standardised rates were computed using the direct method on the basis of the world standard population.

Information on survival is integrated from mortality statistics into the incidence datafile and, for patients who are 'apparently alive', through an active follow-up based on verification of vital status from registries of current residence. The vital status of each case has been verified up to June 30, 1989. Thus, this is one of the few European cancer registries that provides population-based survival data (Levi *et al.*, 1992).

Table I gives the trends in age-standardised (world) rates in two separate age groups. In women aged 30-59, endometrial cancer incidence steadily declined by about 40%, from 20.0/100,000 in 1974-78 to 12.6/100,000 in 1984-88. At older age (≥60 years) no material change in incidence was observed.

Figure 1 presents, on a logarithmic scale, the age curve from 30-39 to 70-79 years in three subsequent calendar quinquennia (1974-78, 1979-83 and 1984-88). The fall in rates was substantial at younger age (i.e., under 50 years, particularly over the last calendar quinquennium) and in middle age (from 50 to 59 years), but no clear pattern of trends was apparent above age 60.

Figure 2 shows survival rates from endometrial cancer in the same three subsequent 5-year calendar periods. Five-year relative survival increased from 0.72 in 1974-78 to 0.77 in 1984-88, and there was a consistent trend of improved survival over more recent calendar periods.

Thus, there are two main findings emerging from this analysis of endometrial cancer trends in the Swiss Canton of Vaud: a decline in incidence, and some improvement (or, in any case, no evidence of worsening) in survival. Trends in survival data are important in order to understand and interpret changes in incidence, since endometrial cancers related to oestrogen replacement treatment have a better prognosis (Robboy & Bradley, 1979), and in the United States a substantial decline in incidence following the fall in oestrogen use has been followed by a worsening in survival rates (Anonymous, 1991).

Following this line of reasoning, changes in pattern of

**Table I** Age-standardised (world) incidence rates for endometrial cancer in two broad age groups. Cancer Registry of Vaud, Switzerland, 1974-1988

| *Calendar period* | *Incidence rates/100,000 women at age:* 30-59 years | ≥60 years | All ages |
|---|---|---|---|
| 1974-78 | 20.0 (108)[a] | 72.2 (203) | 14.6 (313) |
| 1979-83 | 17.0 (95) | 72.4 (221) | 13.4 (316) |
| 1984-88 | 12.6 (72) | 69.0 (208) | 11.7 (280) |
| Average annual population (× 1000) | 108.2 | 34.2 | 142.0 |

[a]Number of cases is given in parentheses.

Correspondence: F. Levi, Registre vaudois des tumeurs, CHUV-Falaises 1, 1011 Lausanne, Switzerland.
Received 1 November 1991; and in revised form 1 May 1992.

**Figure 1** Trends in age-specific (decennial age groups) endometrial cancer incidence in the Canton of Vaud, from 1974 to 1988.

oestrogen replacement use are unlikely to totally explain the declines in endometrial cancer incidence. In the late 1980's, ever use of oestrogen replacement treatment was reported by about 20% of women in peri-menopausal age (Levi *et al.*, 1991). The proportion of ever (mainly past) users aged 60 to 69 was similar (22%), thus indicating that no major change in the prevalence of oestrogen replacement treatment was observed in subsequent generations of Swiss women over the last two decades. Sales data were available from 1985 onwards (IMS—Switzerland, personal communication), and show a modest decline of unopposed oestrogen use. Moreover, there was an appreciable increase in the use of combined oestrogen-progestin treatment, which may have favourably influenced trends in endometrial cancer incidence.

Oral contraceptives started to be used in Switzerland in the late 1960's, and their prevalence of use increased up to the late 1970's. In the late 1980's, the proportion of current users was 25% of women aged 20 to 44 (Levi *et al.*, 1987). In a case-control study conducted in the Canton of Vaud, ever use of oral contraceptives was reported by approximately 60% of women below age 50, and 30% of those aged 50 to 59, and, assuming a protection for ever use of 50% (Levi *et al.*, 1991), this would imply (Bruzzi *et al.*, 1985) an about 20% reduction in incidence under age 60 (i.e., 30% reduction under age 50, and 15% between 50 and 59), whereas rates under age 60 declined by 40%.

There is no reliable information in trends of obesity — the other major established risk factor for endometrial cancer (La Vecchia *et al.*, 1982) — or on the proportion of women with hysterectomy, but it is clear from the data presented that, besides a relevant role of oral contraceptives, and a changed pattern of menopausal replacement treatment use, only a complex of several factors can explain the favourable endometrial cancer trends reported in this Swiss population over the last two decades.

**Figure 2** Relative survival of 909 endometrial cancers according to period of diagnosis. Cancer Registry of Vaud, Switzerland, 1974–88.

### References

ANONYMOUS. (1991). JNCI - News: 1971–1991: diagnosis and treatment advances improve survival. *JNCI*, **83**, 234–236.

BRUZZI, P., GREEN, S.B., BYAR, D.P., BRINTON, L. & SCHAIRER, C. (1985). Estimating the population attributable risk for multiple risk factors using case-control data. *Am. J. Epidemiol.*, **122**, 904–914.

EWERTZ, M. & JENSEN, O.M. (1984). Trends in the incidence of cancer of the corpus uteri in Denmark, 1943–1980. *Am. J. Epidemiol.*, **119**, 725–732.

JICK, H., WALKER, A.M. & ROTHMAN, K.J. (1980). The epidemic of endometrial cancer: a commentary. *Am. J. Public Health*, **70**, 264–267.

LA VECCHIA, C., FRANCESCHI, S., GALLUS, G., DECARLI, A., COLOMBO, E., MANGIONI, C. & TOGNONI, G. (1982). Oestrogens and obesity as risk factors for endometrial cancer in Italy. *Int. J. Epidemiol.*, **11**, 120–126.

LEVI, F. (1987). Statistics from the registry of the Canton of Vaud, Switzerland, 1978–1982. In Muir, C.S., Waterhouse, J., Mack, T., Powell, J. & Whelan, S., (eds.), *Cancer Incidence in Five Continents*, Vol 5, Lyon, International Agency for Research on Cancer, Sci. Publ. No 88, 634–639.

LEVI, F., GUTZWILLER, F., DECARLI, A. & LA VECCHIA, C. (1987). Oral contraceptive use and breast and ovarian cancer mortality in Switzerland. *J. Epidemiol. Commun. Health*, **41**, 267–268.

LEVI, F., MAISONNEUVE, P., FILIBERTI, R., LA VECCHIA, C. & BOYLE, P. (1989). Cancer incidence and mortality in Europe. *Soz. Praeventivmed.*, **34** (Suppl. 2), S1–S84.

LEVI, F., LA VECCHIA, C., GULIE, C., NEGRI, E., MONNIER, V., FRANCESCHI, S., DELALOYE, J.-F. & DE GRANDI, P. (1991). Oral contraceptives and the risk of endometrial cancer. *Cancer Causes and Control*, **2**, 99–103.

LEVI, F., RANDIMBISON, L., TE, V.C., FRANCESCHI, S. & LA VECCHIA, C. (1992). Trends in cancer survival in Vaud, Switzerland. *Eur. J. Cancer*, **28**, 1490–1495.

NISCHAN, P. & EBELING, K. (1991). Endometrial cancer incidence and oral contraception. *Int. J. Epidemiol.*, **20**, 820–821.

PARAZZINI, F., LA VECCHIA, C., BOCCIOLONE, L. & FRANCESCHI, S. (1991). The epidemiology of endometrial cancer. *Gynecol. Oncol.*, **41**, 1–16.

PERSSON, I., SCHMIDT, M., ADAMI, H.-O., BERGSTROM, R., PETTERSSON, B. & SPAREN, P. (1990). Trends in endometrial cancer incidence and mortality in Sweden, 1960–84. *Cancer Causes and Control.*, **1**, 201–208.

ROBBOY, S.J. & BRADLEY, R. (1979). Changing trends and prognostic features in endometrial cancer associated with exogenous estrogen therapy. *Obstet. Gynecol.*, **54**, 269.

VILLARD, L. & MURPHY, M. (1990). Endometrial cancer trends in England and Wales: a possible protective effect of oral contraception. *Int. J. Epidemiol.*, **19**, 255–258.

WALKER, A.M. & JICK, H. (1980). Declining rates of endometrial cancer. *Obstet. Gynecol.*, **56**, 733–736.